# The perplexity of prescribing and switching of biologic drugs in rheumatoid arthritis: a UK regional audit of practice

**DOI:** 10.1186/1471-2474-15-290

**Published:** 2014-09-02

**Authors:** Tim Blake, Vijay Rao, Tahir Hashmi, Nicola Erb, Sheila Catherine O’Reilly, Shireen Shaffu, Karen Obrenovic, Jon Packham

**Affiliations:** Rheumatology Department, Haywood Hospital, High Lane, Burslem, Staffordshire ST6 7AG UK; Rheumatology Department, Birmingham City Hospital, Dudley Road, Birmingham, West Midlands B18 7QH UK; Rheumatology Department, Russells Hall Hospital, Dudley West Midlands, DY1 2HQ UK; Rheumatology Department, Royal Derby Hospital, Uttoxeter Road, Derby, DE22 3NE UK; Rheumatology Department, Leicester Royal Infirmary, Infirmary Square, Leicester, LE1 5WW UK; Clinical Audit Department, Russells Hall Hospital, Dudley, West Midlands DY1 2HQ UK

**Keywords:** Rheumatoid arthritis, Commissioning, Biologic therapies

## Abstract

**Background:**

Biologic drugs are expensive treatments used in rheumatoid arthritis (RA). Switching among them is common practice in patients who have had an inadequate response or intolerable adverse events. The National Institute of Health and Clinical Excellence (NICE) UK, which aims to curtail postcode prescribing, has provided guidance on the sequential prescription of these drugs. This study sought to evaluate the extent to which rheumatology centres across the Midlands were complying with NICE guidance on the switching of biologic drugs in RA, as well as analyse the various prescribing patterns of these drugs.

**Methods:**

Data was collected via a web-based tool on RA patients who had undergone at least one switch of a biologic drug during 2011. The standards specified in NICE technology appraisals (TA130, TA186, TA195, TA198, and TA225) were used to assess compliance with NICE guidance. Descriptive statistical analysis was performed.

**Results:**

There were 335 biologic drug switches in 317 patients. The most common reason given for switching to a drug was NICE guidelines (242, 72.2%), followed by Physician's choice (122, 33.4%). Lack of effect was the most common reason for discontinuing a drug (224, 67%). For patients on Rituximab, Methotrexate was used in 133 switches (76.9% of the time). Overall NICE compliance for all units was 65% (range 50 to 100%), with anti-TNFα to anti-TNFα switches for inefficacy making up the majority of non-compliant switches.

**Conclusion:**

This study draws attention to the enigma and disparity of commissioning and prescribing of biologic drugs in RA. Currently the evidence would not support switching of a biologic drug for non-clinical purposes such as economic pressures. Flexibility in prescribing should be encouraged: biologic therapy should be individualised based on the mode of action and likely tolerability of these drugs. Further work should focus on the evidence for using particular sequences of biologic drugs.

**Electronic supplementary material:**

The online version of this article (doi:10.1186/1471-2474-15-290) contains supplementary material, which is available to authorized users.

## Background

Rheumatoid arthritis (RA) is a chronic systemic inflammatory disease with characteristic pathological changes of synovial hypertrophy, peripheral joint inflammation and destruction, giving the potential for extra-articular manifestations. Prevalence ranges from 0.5-1.5% of the population in industrialised countries, increasing with age and peaking between the ages of 35 and 50 years. The incidence of RA is around 1.5 per 10,000 men and 3.6 per 10,000 women per year. RA is associated with significant morbidity, including pain, disability and work incapacity. Moreover, the disease carries a substantial financial cost to the UK economy, estimated at between £3.8 and £4.75 billion per year [1].

Management of RA consists of an integrated approach that includes both pharmacological and non-pharmacological therapies. Medications include traditional non-biologic and biologic disease-modifying anti-rheumatic drugs (tDMARDs and bDMARDs). Over the past two decades, there has been a major shift in treatment strategy, with emphasis on early introduction of DMARDs, especially the use of biologic agents including anti-tumour necrosis factor alpha (anti-TNFα) agents (Adalimumab, Etanercept, Infliximab etc.) [2–6]. Other classes of bDMARDs comprise B-Cell depleting therapy (Rituximab), Il-6 blocking therapy (Tocilizumab) and T-cell co-stimulatory inhibitor therapy (Abatacept) [7–9]. The efficacy of these agents has been validated in several randomised controlled trials and meta-analyses [10–12]. Switching (or cycling) among biologic therapies has been advocated where patients have shown an inadequate response or adverse events.

The National Institute for Health and Clinical Excellence (NICE) is a UK national health body that was established to reduce postcode prescribing. It has issued disease specific technology appraisals (TAs) 130, 186, 195, 198 and 225, that provide clinicians with guidance on the sequential use of biologic drugs for the treatment of RA [13–17]. In brief, NICE recommends switching between biologic drugs when the first agent (usually an anti-TNFα agent) is associated with an inadequate response or poor tolerability, based on evidence derived primarily from observational studies. Furthermore, a growing body of evidence from randomised controlled trials indicates that the non-TNFα biologic drugs are more effective than placebo in patients with an inadequate response to at least one anti-TNFα agent [7–9, 18].

Biologic agents are expensive treatments when compared to the traditional DMARDs used in rheumatology. Recent economic pressures on healthcare budgets have led to unverified reports of some patients with RA being switched to alternative biologic drugs for non-clinical purposes (such as patient access schemes), despite limited clinical evidence to support this practice.

The aim of this bi-regional audit was to evaluate the extent to which rheumatology units across the Midlands were complying with NICE guidance on the switching of biologic drugs in RA. These units provide rheumatological services to 9.9 million (94.3%) of a total population of 10.5 million people across the Midlands. We also analysed the various prescribing patterns of these drugs across the region, in particular constraints in selecting different agents, and whether switching was being influenced by non-clinical factors.

## Methods

### Study design

Twenty two units across the East and West Midlands were invited to participate in the audit. A questionnaire was sent to lead clinicians at each unit to gather information on the total number of patients with RA on a biologic drug, local restrictions on prescribing biologic drugs, use of first-line biologic agents after failure of conventional DMARDs, and estimation of the number of patients having a 6-monthly 28-joint Disease Activity Score (DAS-28).

An audit proforma was created using the guidance specified in NICE TA130, TA186, TA195, TA198, and TA225, to assess compliance with biologic drug switching in RA. Data was collected on all patients switching from one biologic agent to another during 2011 (Table 1). Data included: age, gender, rheumatoid factor and anti-cyclic citrillunated peptide (anti-CCP) status, sequencing and number of switches of a biologic drug, reasons for choosing to discontinue or start a new biologic drug, drug survival length, use of concomitant Methotrexate (MTX) or other DMARDs at the time of switching, compliance with DAS-28 response criteria, and previous biologic drugs used pre-2011. Patients starting their first biologic agent were excluded from the audit. Some questions allowed for selecting multiple options.

**Table 1:** **Questions and options included in the electronic data collection tool**

1.	Demographic data
	Hospital unit:
	Burton, Cannock, Birmingham City Hospital, Coventry, Derby, Dudley, Hereford, Kings Mill, Leicester, Lincoln, Northampton, Nottingham, Shrewsbury, Solihull, Stoke, University Hospital Birmingham, Warwick, Wolverhampton, Worcester
	Hospital ID number:
	Date of birth:
	Gender:
	Male, female
	Rheumatoid factor status:
	Positive, negative, unknown
	Anti-CCP status:
	Positive, negative, unknown
	How many times did the patient switch (change) their biologic drug in 2011? 1, 2, 3, more than 3
2.	Biologic drug being switched to:
	Abatacept, Adalimumab, Certolizumab, Etanercept, Golilumab, Infliximab, Rituximab, Tocilizumab
3.	Biologic drug being switched from:
	Abatacept, Adalimumab, Certolizumab, Etanercept, Golilumab, Infliximab, Rituximab, Tocilizumab
4.	Why was the biologic drug in 2. chosen?
	Departmental protocol, risk of tuberculosis/other infection, commissioning restriction, patient choice, research participation, pharmaceutical incentive, physician’s choice
5.	Why was the biologic drug in 3. discontinued?
	End of trial, lack of effect, intolerance/adverse event, drug site reaction, cardiac side effects, respiratory side effects, malignancy, neuropathy, demyelination, tuberculosis, septic arthritis, other infection
6.	Did the patient continue with the biologic drug in 2. for the next six months?
	Yes, no
	If Yes, did the DAS-28 score meet NICE response criteria?
	Yes, no, not assessed
7.	Was concomitant Methotrexate used?
	Yes, No
	If No, what was the reason for not using?
	Free text
8.	Other concomitant DMARDs used at the time of the drug switch:
	Azathioprine, Ciclosporin, Corticosteroids, Cyclophosphamide, Gold, Hydroxychloroquine, Leflunomide, Penicillamine, Sulfasalazine, Other
9.	Repeat steps 2. To 8. with successive switches
10.	Biologic drugs used pre-2011:
	Abatacept, Adalimumab, Certolizumab, Etanercept, Golilumab, Infliximab, Rituximab, Tocilizumab

### Ethics

The survey was reviewed by the local Research and Development team and was deemed not to require Ethics Committee approval.

### Audit standards

Standards were derived from the NICE commissioning algorithm on biologic drugs in RA, that has consolidated guidance from the five technology appraisals (TAs 130, 186, 195, 198 and 225) on the use of biologic drugs based on efficacy, safety and cost-effectiveness.

The current standard of practice in the UK is to start a patient on anti-TNFα therapy when they demonstrate a DAS-28 > 5.1 on two occasions, one month apart, and failure of two tDMARDs (one being MTX).

The decision to initiate a biologic drug should be guided by whether the patient is taking MTX. Where MTX is co-prescribed, NICE instructs the clinician to use the least expensive TNFα inhibitor: Adalimumab + MTX (TA 130); Certolizumab + MTX (TA 186); Etanercept + MTX (TA 130); Golimumab + MTX (TA 225); or Infliximab + MTX (TA 130). If a biologic drug has to be withdrawn because of an adverse event within the first six months of treatment, initiation of an alternative TNFα inhibitor may be considered.

For patients displaying a suboptimal response to anti-TNFα therapy (defined as a lack of improvement in DAS-28 of 1.2 or more at six months), clinicians may consider switching to a biologic agent in a different drug class. At the end of 2011, the only second-line biologic drug recommended for this purpose was Rituximab, to be co-prescribed with MTX (TA 195).

If Rituximab has to be withdrawn because of an adverse event, one may consider treatment with the following: Abatacept + MTX (TA 195); Adalimumab + MTX (TA 195); Etanercept + MTX (TA 195); Golimumab + MTX (TA 225); Infliximab + MTX (TA 195); or Tocilizumab + MTX (TA 198).

Inadequate responders to Rituximab (defined as a lack of improvement in DAS-28 of 1.2 or more at six months), are directed to Tocilizumab, to be co-prescribed with MTX (TA 198).

In the case of intolerance to MTX, NICE permits the use of Adalimumab (TA 130), Etanercept (TA 130) and Certolizumab (TA 186) as monotherapy, in accordance with drug licenses. An alternative TNFα inhibitor in this group may be considered, if there is an adverse event within the first six months of treatment requiring cessation of the original biologic agent.

### Data analysis

Data was collected via web-based data collection software hosted at one of the participating audit centres (Russells Hall Hospital, Dudley) and was subsequently exported into Microsoft Excel Descriptive data analysis was performed using Microsoft excel and PASW® Statistics 18.

## Results

Eighteen of the 22 invited units participated in the audit; each one was randomly issued with a participating number. There were 335 biologic drug switches in a total of 317 patients across the 18 participating rheumatology units. Forty-five percent (143/317) of patients were in the age range of 51 to 65 (mode). Seventy-five percent (238/317) of patients were female. Sixty-seven percent (212/317) of patients displayed seropositivity for rheumatoid factor and/or anti-CCP antibodies. In 94% (299/317) of cases, switching of biologic drugs occurred only once during the 12 month study period.

### Selection of biologic drugs

Of the switches, 85.7% (287/335) were from an anti-TNFα agent: Etanercept 38.5% (129), Adalimumab 24.2% (81), Certolizumab 15.2% (51), and Infliximab 7.8% (26). 13.4% (45/335) were from Rituximab, and 0.9% (3/335) from Tocilizumab. There were no switches from Abatacept.

One hundred and seventy three of 335 (51.6%) switches were to Rituximab, with the majority being from an anti-TNFα agent (99.4%, 172/173). Similarly, 29.6% (99/335) of switches were to an anti-TNFα agent (42.4% (42) to Etanercept, 31.3% (31) to Adalimumab, 19.2% (19) to Certolizumab, 5.1% (5) to Infliximab and 2.0% (2) to Golimumab. The overall sequencing of biologic drug switches is depicted in Table 2.

**Table 2:** **Overall sequencing of biologic drug switches**

	Anti-TNFα	Rituximab	Tocilizumab	Abatacept	Total
	n (%)	n (%)	n (%)	n (%)	n (%)
Anti-TNFα	84 (25.1)	172 (51.3)	21 (6.3)	10 (3.0)	**287 (85.7)**
n (%)					
Rituximab	15 (4.4)	-	26 (7.8)	4 (1.2)	**45 (13.4)**
n (%)					
Tocilizumab	0 (0)	1 (0.3)	-	2 (0.6)	**3 (0.9)**
n (%)					
Abatacept	0 (0)	0 (0)	0 (0)	-	**0 (0)**
n (%)					
**Total**	**99 (29.6)**	**173 (51.6)**	**47 (14.0)**	**16 (4.8)**	**335 (100)**
**n (%)**					

The most common reason given for switching was NICE guidelines (72.2%, 242/335), followed jointly by Physician’s choice (33.4%, 122/335) and Departmental protocol (33.4%, 122/335). Pharmaceutical incentives made up 0.3% (1/335) of reasons for switching of a biologic drug. A proportion of switches were for more than one reason. Lack of effect was the most common reason cited for discontinuing a drug (67%, 224/335), followed by adverse drug reactions (22.7%, 76/335).

### Rituximab (first-line biologic) therapy

For patients on Rituximab, Methotrexate was used 76.9% (133/173) of the time, and 67.1% (116/173) of patients who were switched to Rituximab were seropositive. With regards to reasons cited for not using Methotrexate in this population, 75% (30/40) had previous intolerance to the drug, 10% (4/40) had previous inefficacy, 5% (2/40) had documented lung disease that would preclude treatment, 2% (1/40) stated patient choice, and in 8% (3/40) no reason was given. Where Rituximab was used without Methotrexate, other tDMARDs were used in 47.5% (19/40) (some in combination): Leflunomide 20.0% (8/40); Sulfasalazine 20.0% (8/40); Corticosteroids 7.5% (3/40); Hydroxychloroquine 2.5% (1/40), Gold 2.5% (1/40) and Ciclosporin 2.5% (1/40). 52.5% (21/40) were switched to Rituximab without concomitant tDMARD usage.

### Monitoring

In 76% (253/335) of switches, the drug was continued for six months. Of these, 80% (202/253) had DAS-28 response criteria measured at NICE recommended time points.

### Compliance with NICE guidance

**Figure 1:**
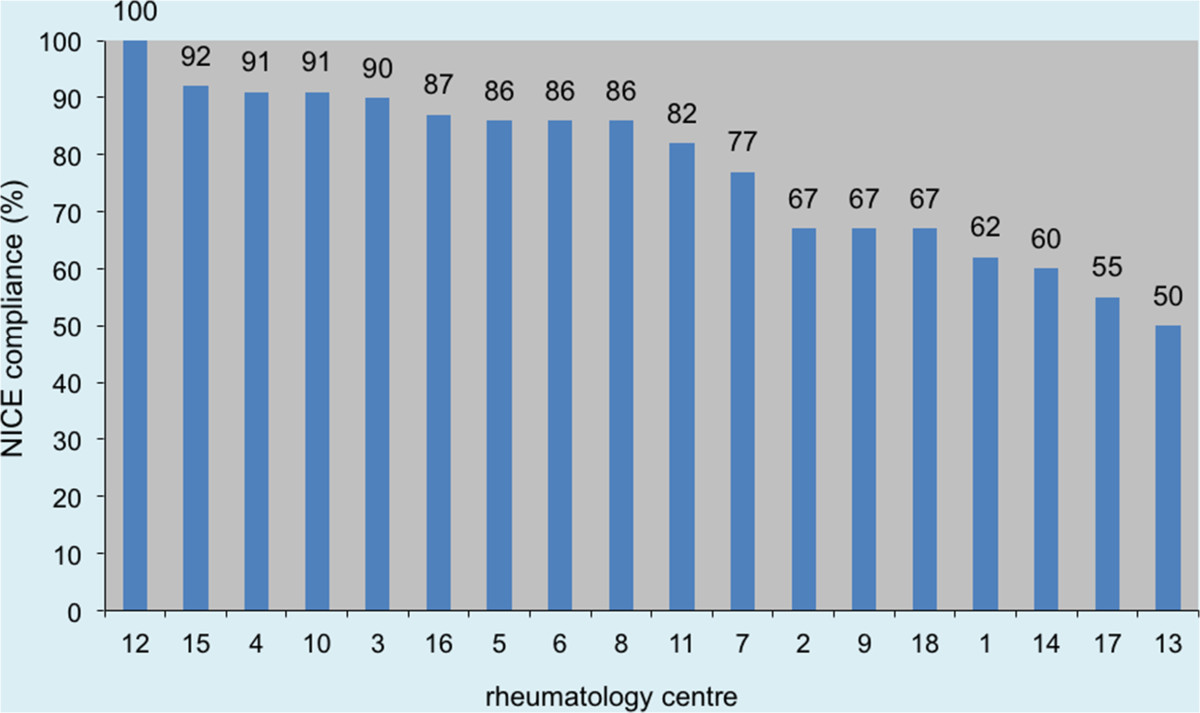
**Overall comparison of NICE compliance for each rheumatology unit.**

Referring to NICE guidance during 2011, NICE compliance for all units was 65% (range 50-100%), as depicted in Figure 1. The use of Rituximab without Methotrexate made up the majority of non-compliant switching (34%, 40/116). This was followed by anti-TNFα to anti-TNFα switching for inefficacy (28%, 32/116); anti-TNFα switching to Tocilizumab prior to Rituximab (14%, 16/116); Rituximab switching to anti-TNFα in cases of inadequate response (12%, 14/116); anti-TNFα switching to Abatacept (9%, 10/116); and Rituximab to Abatacept (3%, 4/116).

**Figure 2:**
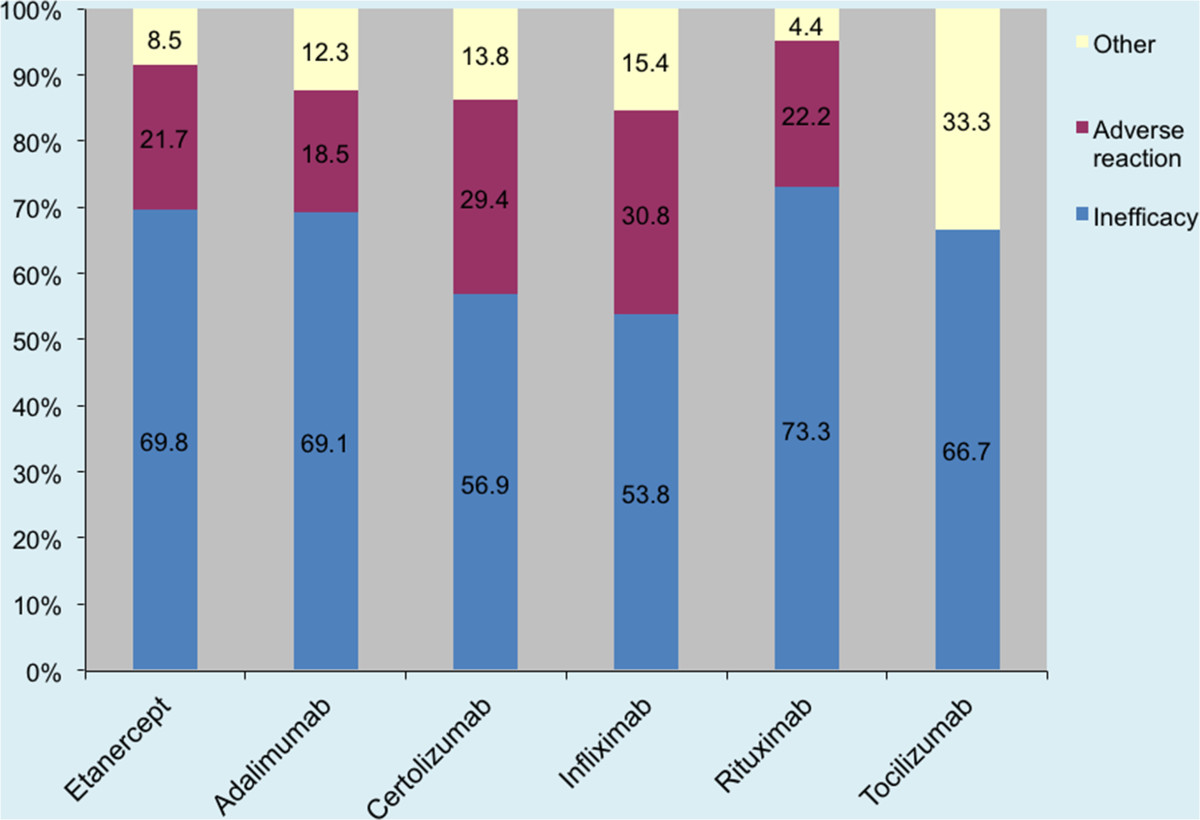
**Reasons for switching from a biologic drug.**

**Figure 3:**
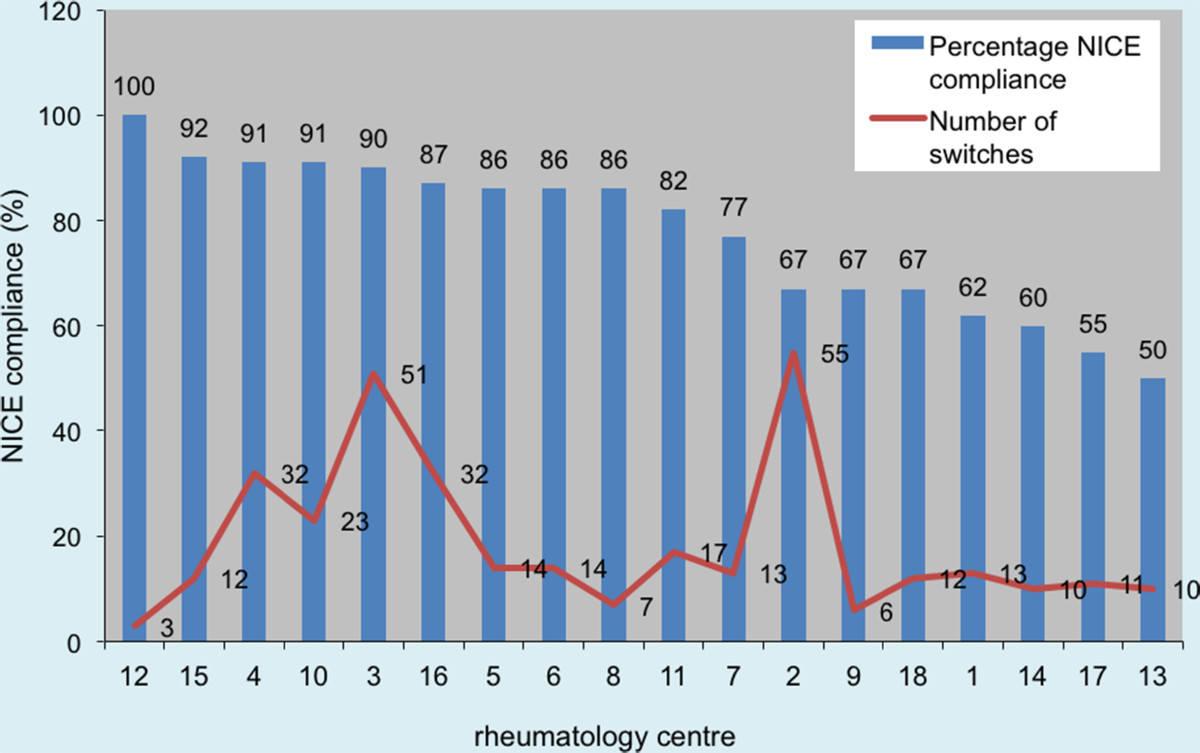
**Number of switches compared to NICE compliance.**

**Figure 4:**
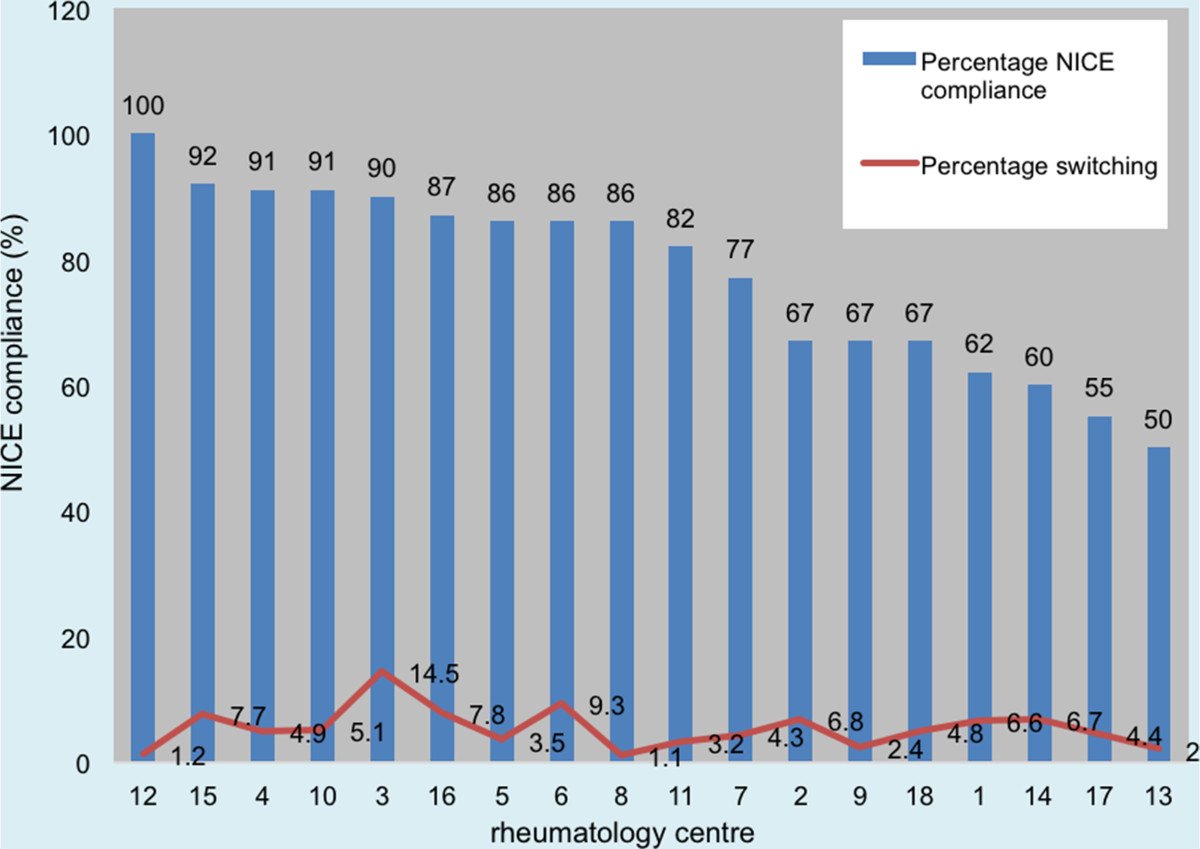
**Percentage switching compared to NICE compliance.**

**Figure 5:**
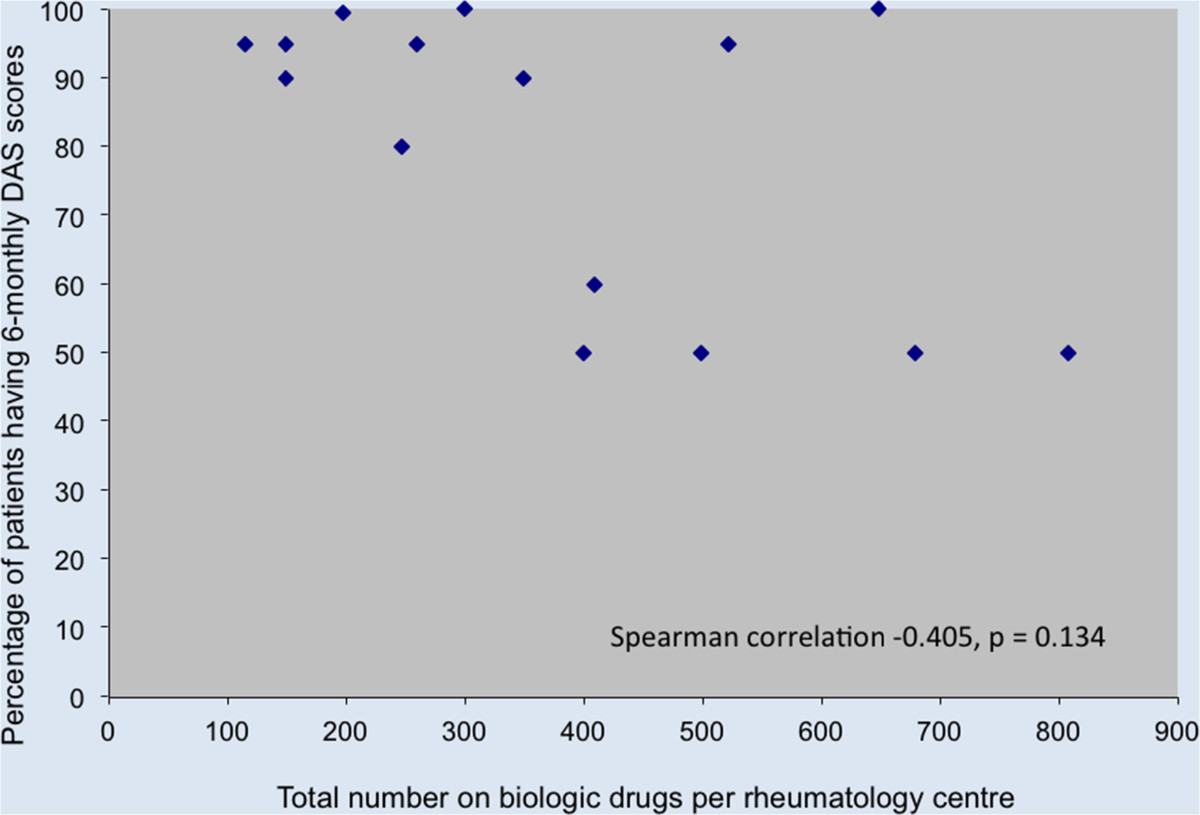
**DAS-28 compliance compared to the total number of patients on biologic drugs.**

Subsequent alteration to NICE guidance since 2011 allows anti-TNFα switching to Tocilizumab, and Abatacept prior to Rituximab for an adverse event. This comprises 46.6% of causes for failure of compliance in our 2011 data.Figure 2 illustrates the reasons for switching among different biologic drugs. More patients switched from Certolizumab (29.4%, 15/51) and Infliximab (30.7%, 8/26) due to adverse reactions. With respect to inefficacy, more patients were switched from Rituximab (73%, 33/45) than any other biologic drug.Our results demonstrate disparity in prescribing patterns between individual rheumatology centres, in terms of the number of switches and percentage of patients on a biologic drug being switched (number of switches/total number of patients on a biologic drug × 100 per unit). Figures 3 and 4 compare these factors against NICE compliance for individual units.There is a negative correlation between the number of patients on a biologic drug and the percentage of patients having six-monthly DAS-28 assessments in each rheumatology centre (Figure 5).

## Discussion

In general, this audit, which represents over 95% of the population of the East and West Midlands, shows that most rheumatology centres are broadly compliant with NICE guidance on the switching of biologic drugs in their RA patients. As would be expected, biologic drugs that were being switched included well-established medications, which still comprise the majority of biologic use within rheumatological practice (such as Infliximab and Etanercept). The biologic treatments that are being switched to, include a much broader range of therapeutic options, including more recently NICE endorsed drugs, such as Tocilizumab and Abatacept. The majority of biologic switches related to inefficacy, with less than a quarter being related to drug related side effects/ intolerance. This audit has shown that anti TNFα to anti TNFα switching for inefficacy is one of the commonest causes of non-compliance with NICE guidance. This finding has some relevance when planning potential biologic treatment flows, as having intolerance to individual therapies appears to allow a wider selection of therapeutic options with the NICE framework when compared to inefficacy.

This audit highlights a wide variation in compliance between individual centres with NICE guidelines on the switching of biologic drugs in RA (between 50 and 100%). This suggests that there are major local influences on compliance. Although this could be related to clinicians being unaware or unwilling to follow NICE guidance, it is likely that certain rheumatology units have local agreements that permit prescription outside these constraints. However, with such complex and changeable guidelines for RA treatment, it would not be surprising to find confusion regarding best practise in this area due to: a) conflicting national and international guidelines [19], b) updates in NICE guidance and c) changes in the scientific evidence base. Recent NICE guidance, which offers a wider spectrum of first-line biologic treatment options, may help this situation, although the stipulation of using the ‘cheapest option’ may have a contrary effect, restricting the potential for individualised care.

There is increasing fragmentation of commissioning with the introduction of clinical commissioning groups (CCGs), which has the potential to worsen inequality and disparity between geographical areas, by the application of complex drug pathways. The audit raises important questions about how these drugs should be commissioned in the future, particularly with the advent of specialist commissioning structures now taking a leading role in advising on biologic use outside of NICE guidance for certain rheumatological diseases (such as SLE).

The major areas of suboptimal adherence pertained to sequencing of biologic drugs in relation to inadequate response (which is the most likely area to be influenced by new scientific evidence), use of Rituximab without MTX, and monitoring of therapy. We know from phase II studies that co-administration of Rituximab and MTX affords a better outcome [20], with only limited evidence for co-administration of other tDMARDs. The extent to which the benefit of MTX influences the efficacy of newer biologic agents remains to be fully defined and is the subject of on-going interventional studies. For the present time, clinicians should generally aim to use these agents in conjunction with MTX. The significant number of patients within this audit that received Rituximab in the absence of methotrexate highlights the importance of further research into this area.

There is a non-significant trend towards a negative correlation between the number of RA patients in any one centre on a biologic drug and the proportion of those having regular DAS-28 assessments. This finding may suggest that a strain on existing resources may adversely affect patient surveillance. This is an important factor to consider when developing and commissioning future rheumatology services, particularly as departments merge and biologic drug use escalates. Although DAS-28 has its limitations, it will remain the main biologic response assessment tool, until a validated alternative can be developed [21]. Local and national commissioning of biologic therapy clearly needs to take into consideration support services for drug monitoring as well as the not so insignificant drug costs.

Currently the evidence would not support switching of a biologic drug for non-clinical purposes. Despite significant financial strain on the NHS, it was encouraging to see that financially driven switches in biologic medication occur, but are extremely rare. Nevertheless, there is likely to be a reluctance to declare pharmaceutical incentives as a reason for choosing a particular biologic drug.

The prevailing limitation of this study is the fact that it is impossible to state the overall efficacy and tolerability of these drugs as only patients who had switched treatment were included. In addition, the rheumatoid cohort studied reflected a more diverse and heterogeneous autoantibody profile than the standard RA population quoted in the literature [22]. This suggests that this cohort may not simply consist of classical rheumatoid arthritis patients, but may include some individuals with less well-defined inflammatory arthritis. This audit does not provide particularly useful data on side effect profiles of individual biologic therapies, as it did not include all patients on biologic treatment under rheumatological care.

The results of this survey, including comparative data in an anonymised form, have been disseminated to individual units, and presented and discussed at regional meetings.

Further work should be done to determine the evidence for using particular sequences of biologic drugs in RA patients who are either intolerant or inadequate responders.

## Conclusion

This regional survey not only provides us with important regional data on NICE compliance regarding switching of biologic drug therapy in RA, but it also informs us of the wide variance in prescribing practice of these drugs. Using Rituximab without MTX and anti-TNFα to anti-TNFα switching due to inefficacy were the main reasons for non-compliance. We suggest that individual units assess how well NICE guidance has been implemented and adhered to at local level to assess the cost impact. Use of national commissioning for all, and development of a common but flexible biologic pathway may facilitate smoother and more consistent funding for these drugs in the future. Flexibility in prescribing is important, as biologic therapy should be individualised based on the mode of action and likely tolerability of these drugs.

## Authors’ information

TB is a Specialist Registrar trainee in Rheumatology and General Internal Medicine in the West Midlands Deanery, and also undertaking a Masters in Medical Education (MMedEd) at The University of Warwick. VR, TH and SS are Specialist Registrar trainees in Rheumatology at the time of conducting this study. NE, SOR and NE are practising Consultants in Rheumatology. KO is responsible for leading on several local and regional audit projects.

## Authors’ original submitted files for images

Below are the links to the authors’ original submitted files for images. Authors’ original file for figure 1 Authors’ original file for figure 2 Authors’ original file for figure 3 Authors’ original file for figure 4 Authors’ original file for figure 5 Authors’ original file for figure 6

## References

[1] National Institute for Health and Clinical Excellence (NICE) Clinical Guideline 79. Rheumatoid arthritis: The Management of Rheumatoid Arthritis in Adults.http://www.nice.org.uk/nicemedia/live/12131/43327/43327.pdf,

[2] Smolen JS, Aletaha D, Bijlsma JWJ, Breedveld FC, Boumpas D, Burmester G, Combe B, Cutolo M, de Wit M, Dougados M, Emery P, Gibofsky A, Gomez-Reino JJ, Haraoui B, Kalden J, Keystone EC, Kvien T, McInnes I, Martin-Mola E, Montecucco C, Schoels M, van der Heijde D, T. T. Expert Comm (2010). Treating rheumatoid arthritis to target: recommendations of an international task force. Ann Rheum Dis.

[3] Edwards JCW, Leandro MJ (2004). B lymphocyte depletion therapy with rituximab in rheumatoid arthritis. Rheum Dis Clin North Am.

[4] Kremer JM, Westhovens R, Leon M, Di Giorgio E, Alten R, Steinfeld S, Russell A, Dougados M, Emery P, Nuamah IF, Williams GR, Becker JC, Hagerty DT, Moreland LW (2003). Treatment of rheumatoid arthritis by selective inhibition of T-cell activation with fusion protein CTLA4Ig. N Engl J Med.

[5] Maini RN, Taylor PC, Pavelka K, Emery P, Szechinski J, Balint G, Broll J, Grp CS (2003). Efficacy of IL-6 receptor antagonist Mra in rheumatoid arthritis patients with an incomplete response to methotrexate (CHARISMA). Arthritis Rheum.

[6] Kiely PDW, Brown AK, Edwards CJ, O’Reilly DT, Ostor AJK, Quinn M, Taggart A, Taylor PC, Wakefield RJ, Conaghan PG (2009). Contemporary treatment principles for early rheumatoid arthritis: a consensus statement. Rheumatology.

[7] Cohen SB, Emery P, Greenwald MW, Dougados M, Furie RA, Genovese MC, Keystone EC, Loveless JE, Burmester G-R, Cravets MW, Hessey EW, Shaw T, Totoritis MC, Reflex Trial Group (2006). Rituximab for rheumatoid arthritis refractory to anti-tumor necrosis factor therapy - results of a multicenter, randomized, double-blind, placebo-controlled, phase III trial evaluating primary efficacy and safety at twenty-four weeks. Arthritis Rheum.

[8] Emery P, Keystone E, Tony HP, Cantagrel A, van Vollenhoven R, Sanchez A, Alecock E, Lee J, Kremer J (2008). IL-6 receptor inhibition with tocilizumab improves treatment outcomes in patients with rheumatoid arthritis refractory to anti-tumour necrosis factor biologicals: results from a 24-week multicentre randomised placebo-controlled trial. Ann Rheum Dis.

[9] Genovese MC (2005). Abatacept for rheumatoid arthritis refractory to tumor necrosis factor alpha inhibition (vol 353, pg 1114, 2005). N Engl J Med.

[10] Singh JA, Christensen R, Wells GA, Suarez-Almazor ME, Buchbinder R, Lopez-Olivo MA, Ghogomu ET, Tugwell P (2009). Biologics for rheumatoid arthritis: an overview of Cochrane reviews. Cochrane Database Syst Rev.

[11] Keystone EC, Kavanaugh AF, Sharp JT, Tannenbaum H, Hua Y, Teoh LS, Fischkoff SA, Chartash EK (2004). Radiographic, clinical, and functional outcomes of treatment with adalimumab (a human anti-tumor necrosis factor monoclonal antibody) in patients with active rheumatoid arthritis receiving concomitant methotrexate therapy - A randomized, placebo-controlled, 52-week trial. Arthritis Rheum.

[12] Klareskog L, van der Heijde D, de Jager JP, Gough A, Kalden J, Malaise M, Mola EM, Pavelka K, Sany J, Settas L, Wajdula J, Pedersen R, Fatenejad S, Sanda M, Tempo Study Investigators (2004). Therapeutic effect of the combination of etanercept and methotrexate compared with each treatment alone in patients with rheumatoid arthritis: double-blind randomised controlled trial. Lancet.

[13] NICE TA 130.http://www.nice.org.uk/nicemedia/live/11867/37914/37914.pdf,

[14] NICE TA 186.http://www.nice.org.uk/nicemedia/live/12808/47544/47544.pdf,

[15] NICE TA 195.http://www.nice.org.uk/nicemedia/live/13108/50413/50413.pdf,

[16] NICE TA 198.http://www.nice.org.uk/nicemedia/live/13669/58202/58202.pdf,

[17] NICE TA 225.http://www.nice.org.uk/nicemedia/live/13490/54929/54929.pdf,

[18] Emery P, Sarzi-Puttini P, Moots RJ, Andrianakos A, Sheeran TP, Choquette D, Finckh A, Desjuzeur ML, Gemmen EK, Mpofu C, Gottenberg JE (2012). Relative efficacy of Rituximab versus an alternative TNF inhibitor in patients with rheumatoid arthritis and an inadequate response to a single previous TNF inhibitor: interim results from SWITCH-RA, a global, observational, comparative effectiveness study. Rheumatology.

[19] Smolen JS, Landewe R, Breedveld FC, Dougados M, Emery P, Gaujoux-Viala C, Gorter S, Knevel R, Nam J, Schoels M, Aletaha D, Buch M, Gossec L, Huizinga T, Bijlsma J, Burmester G, Combe B, Cutolo M, Gabay C, Gomez-Reino J, Kouloumas M, Kvien TK, Martin-Mola E, McInnes I, Pavelka K, van Riel P, Scholte M, Scott DL, Sokka T, Valesini G (2010). EULAR recommendations for the management of rheumatoid arthritis with synthetic and biological disease-modifying antirheumatic drugs. Ann Rheum Dis.

[20] Emery P, Fleischmann R, Filipowicz-Sosnowska A, Schechtman J, Szczepanski L, Kavanaugh A, Racewicz AJ, van Vollenhoven RF, Li NF, Agarwal S, Hessey EW, Shaw TM, Dancer Study Grp (2006). The efficacy and safety of rituximab in patients with active rheumatoid arthritis despite methotrexate treatment - Results of a phase IIb randomized, double-blind, placebo-controlled, dose-ranging trial. Arthritis Rheum.

[21] Deighton C, Hyrich K, Ding T, Ledingham J, Lunt M, Luqmani R, Kiely P, Bukhari M, Abernethy R, Ostor A, Bosworth A, Gadsby K, McKenna F, Finney D, Dixey J (2010). BSR and BHPR rheumatoid arthritis guidelines on eligibility criteria for the first biological therapy (vol 49, pg 1197, 2010). Rheumatology.

[22] Nishimura K, Sugiyama D, Kogata Y, Tsuji G, Nakazawa T, Kawano S, Saigo K, Morinobu A, Koshiba M, Kuntz KM, Kamae I, Kumagai S (2007). Meta-analysis: diagnostic accuracy of anti-cyclic citrullinated peptide antibody and rheumatoid factor for rheumatoid arthritis. Ann Intern Med.

[CR23] The pre-publication history for this paper can be accessed here:http://www.biomedcentral.com/1471-2474/15/290/prepub

