# Supplementary figures and images for: The perplexity of prescribing and switching of biologic drugs in rheumatoid arthritis: a UK regional audit of practice
Source: BMC Musculoskelet Disord. 2014 Sep 2;15:290. doi: 10.1186/1471-2474-15-290 (PMC4164745; doi:10.1186/1471-2474-15-290)

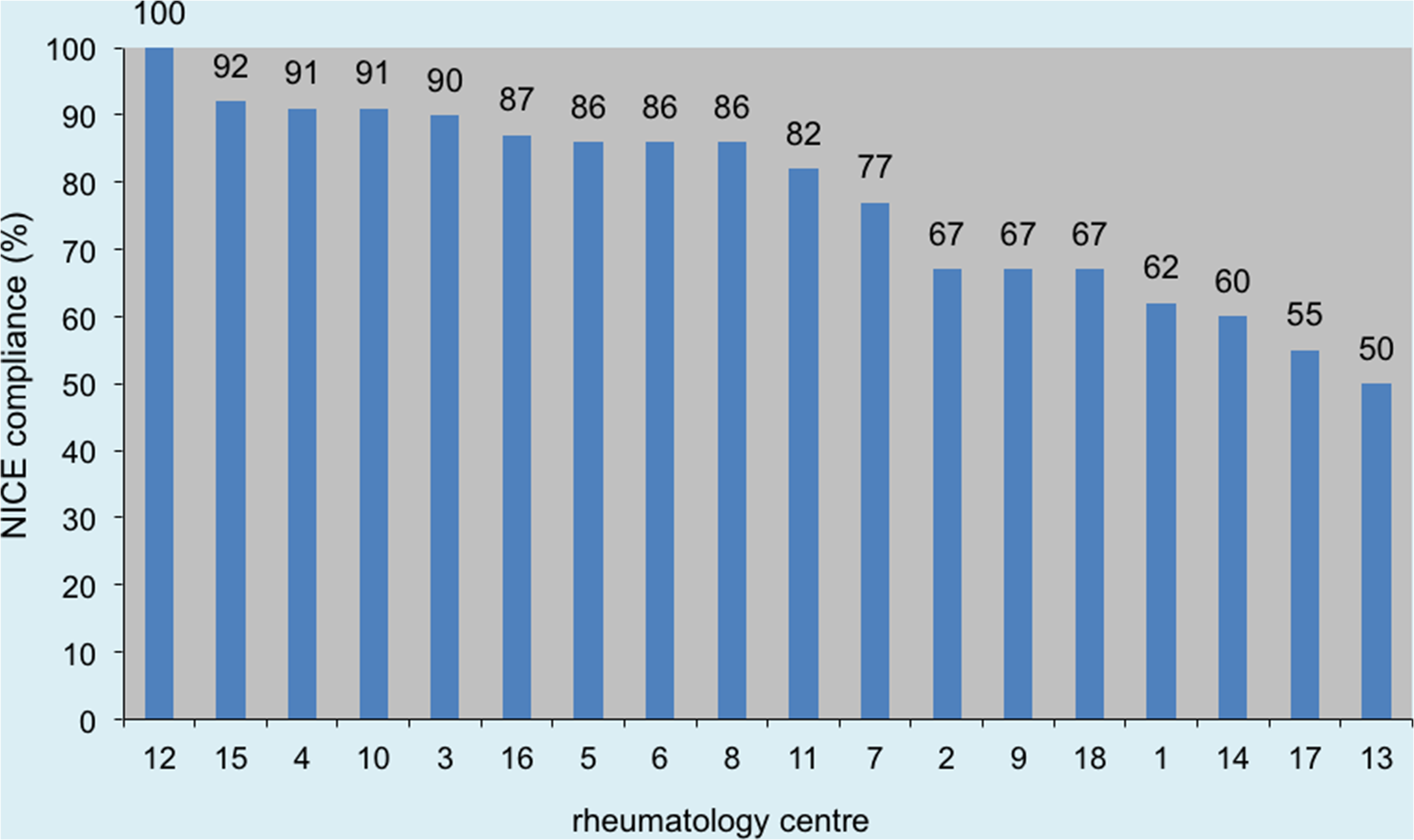

Supplement: Supplementary file 1 — Authors’ original file for figure 1 [file 12891_2014_2240_MOESM1_ESM.tiff]

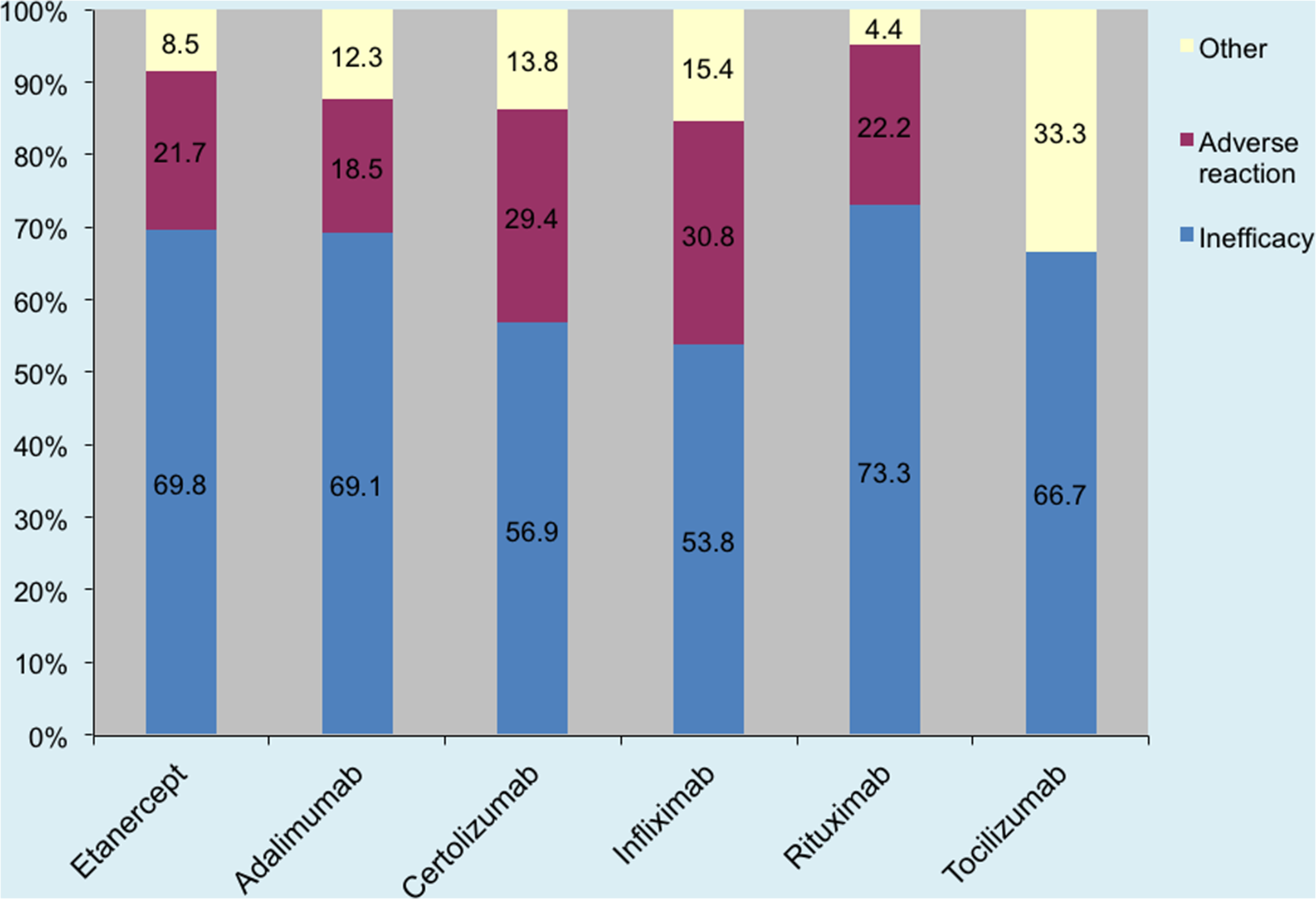

Supplement: Supplementary file 2 — Authors’ original file for figure 2 [file 12891_2014_2240_MOESM2_ESM.tif]

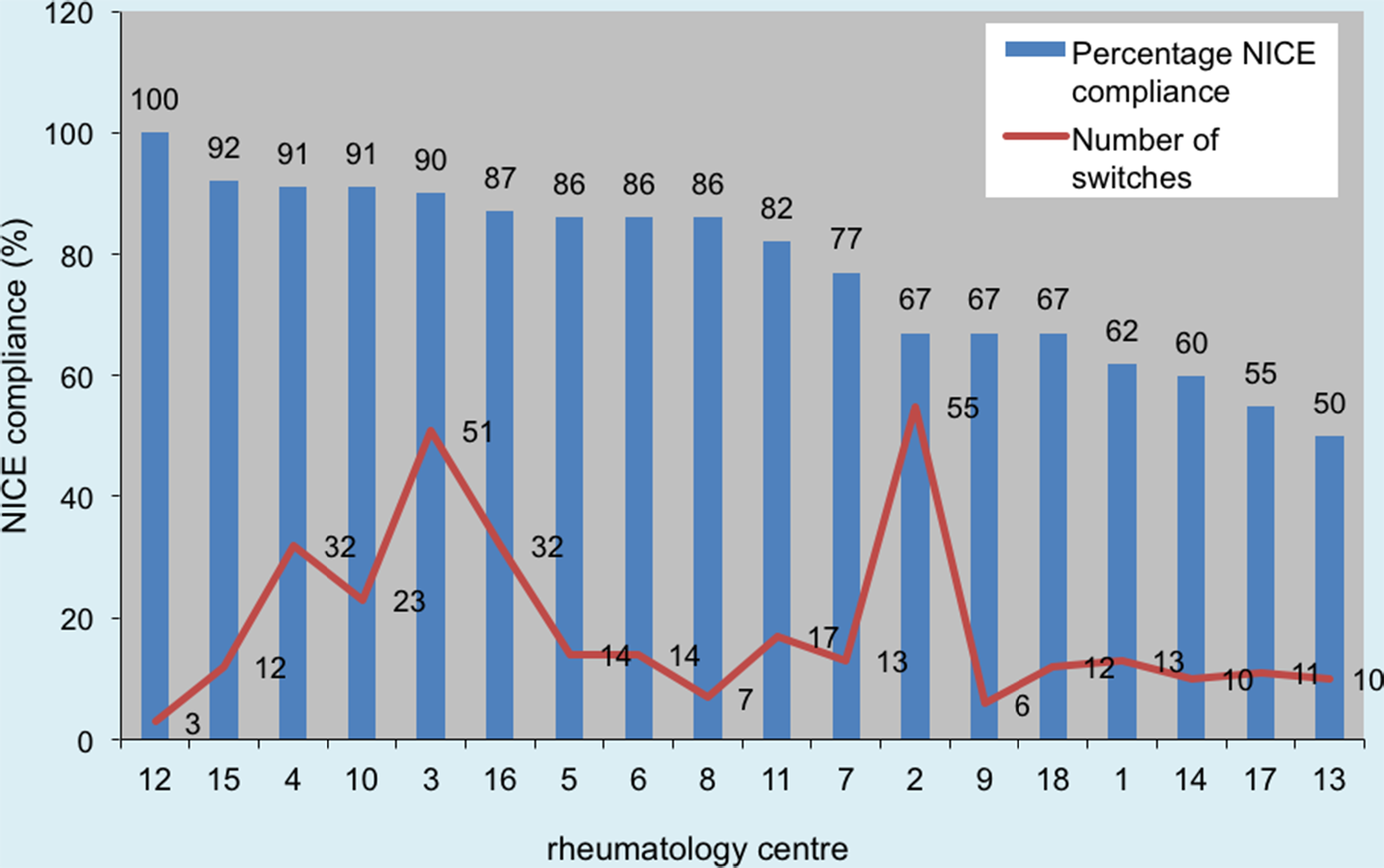

Supplement: Supplementary file 3 — Authors’ original file for figure 3 [file 12891_2014_2240_MOESM3_ESM.tif]

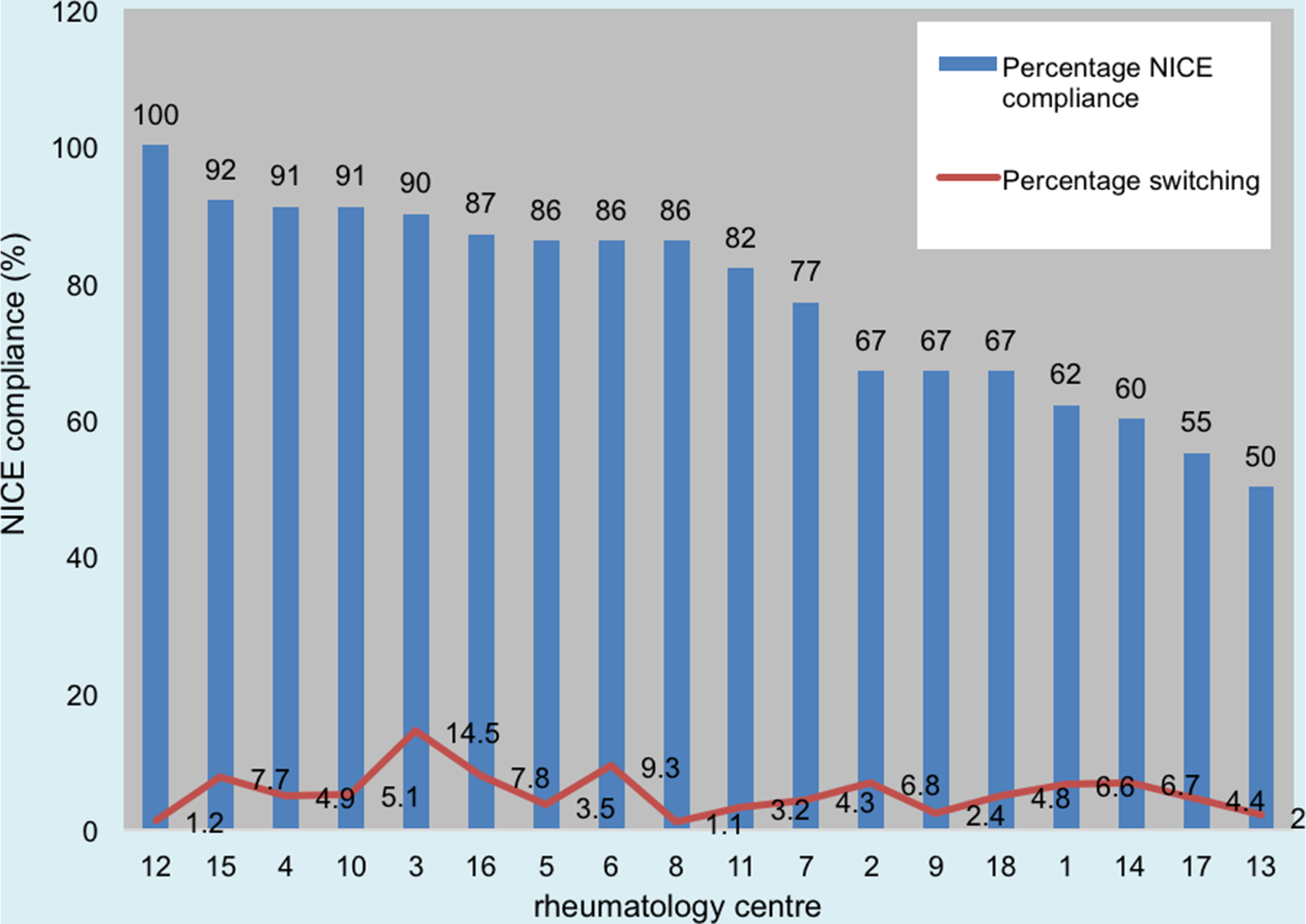

Supplement: Supplementary file 4 — Authors’ original file for figure 4 [file 12891_2014_2240_MOESM4_ESM.tif]

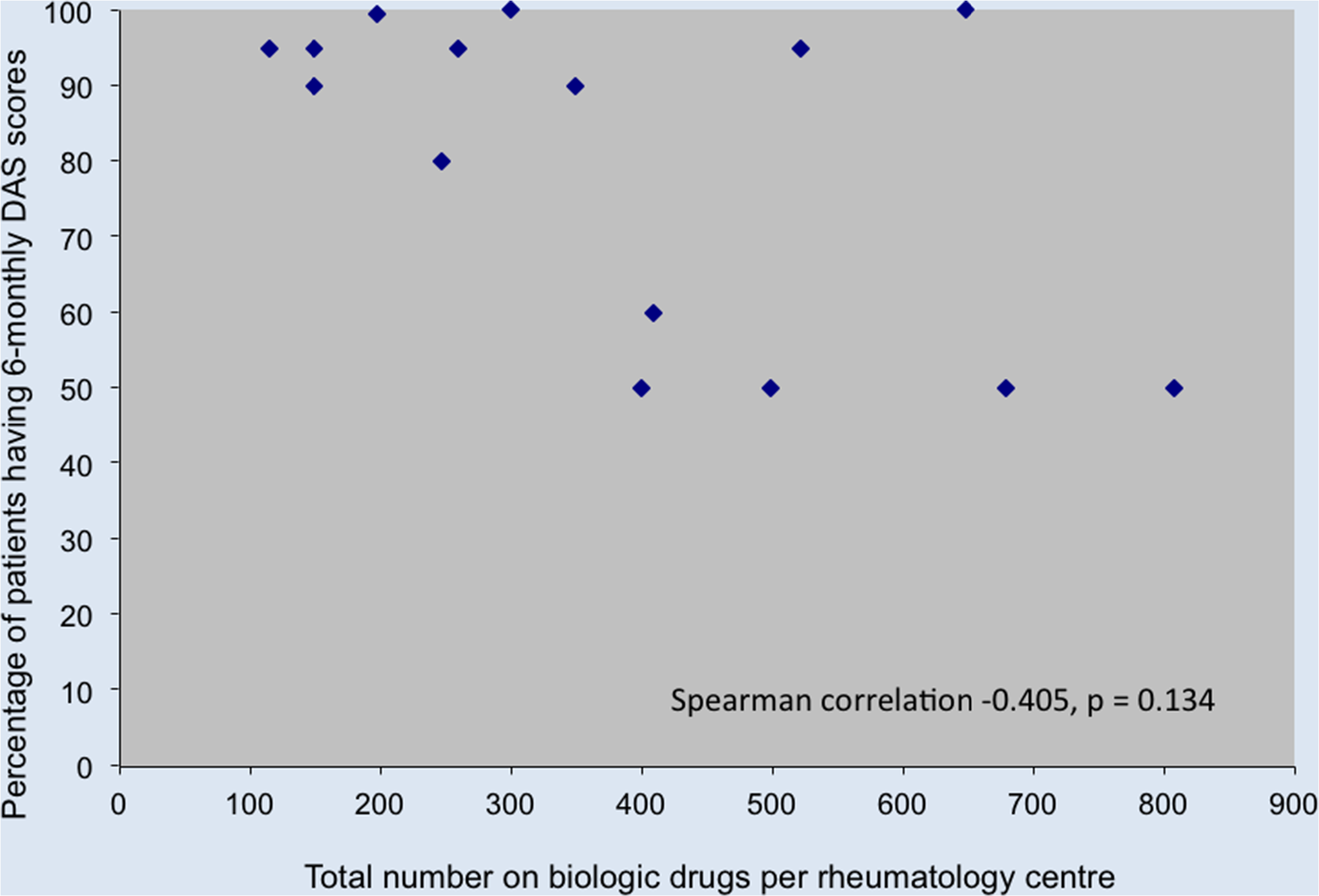

Supplement: Supplementary file 5 — Authors’ original file for figure 5 [file 12891_2014_2240_MOESM5_ESM.tif]

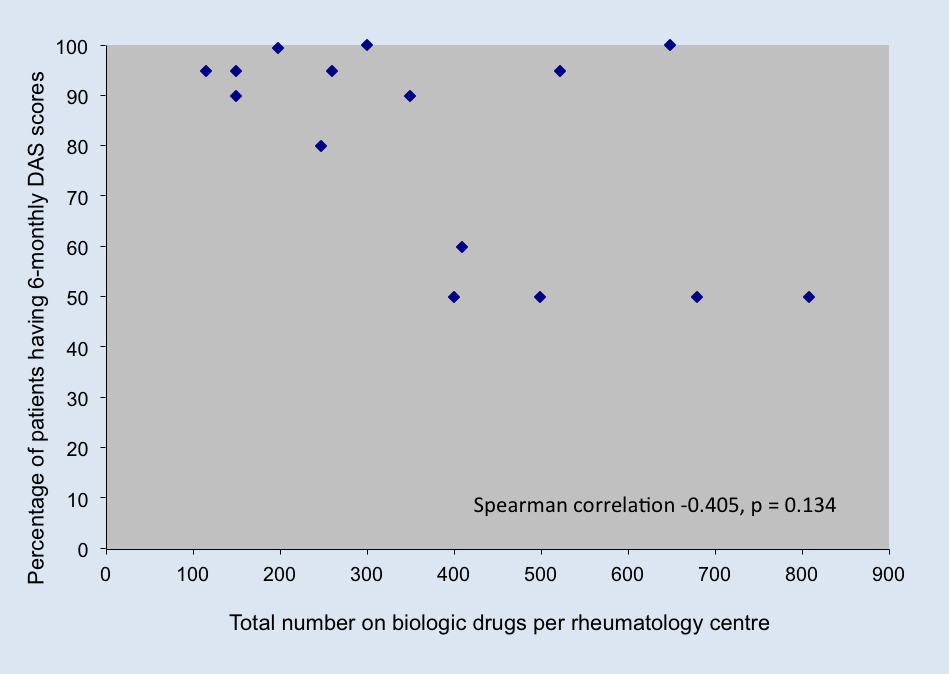

Supplement: Supplementary file 6 — Authors’ original file for figure 6 [file 12891_2014_2240_MOESM6_ESM.tiff]
